# Is the Cooling Vest an Ergogenic Tool for Physically Active Individuals? Assessment of Perceptual Response, Thermo-Physiological Behavior, and Sports Performance: A Systematic Review and Meta-Analysis

**DOI:** 10.3390/bioengineering10020132

**Published:** 2023-01-18

**Authors:** Diego Fernández-Lázaro, Juan F. García, Luis Antonio Corchete, Miguel Del Valle Soto, Gema Santamaría, Jesús Seco-Calvo

**Affiliations:** 1 Department of Cellular Biology, Genetics, Histology and Pharmacology, Faculty of Health Sciences, Campus of Soria, University of Valladolid, 42004 Soria, Spain; 2 Neurobiology Research Group, Faculty of Medicine, University of Valladolid, 47005 Valladolid, Spain; 3 Department of Mechanical, Informatics and Aerospatiale Engineering, University of Leon, 24071 Leon, Spain; 4 Network Center for Biomedical Research in Cancer (CIBERONC), 37007 Salamanca, Spain; 5 Department of Cellular Morphology and Biology, University of Oviedo, 33006 Oviedo, Spain; 6 Department of Anatomy and Radiology, Faculty of Health Sciences, Campus of Soria, University of Valladolid, 42004 Soria, Spain; 7 Physiotherapy Department, Institute of Biomedicine (IBIOMED), Campus de Vegazana, University of Leon, 24071 León, Spain; 8 Physiology Department, Faculty of Medicine, Basque Country University, 48900 Leioa, Spain

**Keywords:** cooling vest, ergogenic tool, heat conditions, thermo-physiology behavior, perceptual response, sports performance

## Abstract

Exercise capacity is limited by environmental heat stress because thermoregulatory systems are altered and cannot prevent the elevation of body temperature due to a complex interplay of physiological, physical, and perceptual alterations. Cooling is an effective strategy to attenuate the temperature rise. Based on the Preferred Reporting Items for Systematic Reviews and Meta-Analyses (PRISMA) guidelines and the PEDro scale for assessing methodological quality, we systematically reviewed studies indexed in Medline, Web of Science, EMBASE, Science Direct, Sportdiscus, and Scopus, to evaluate the effects of the cooling vest (CVs) on perceptual response, physiological behavior, and sports performance in adult physical activity practitioners under heat stress conditions. Among the 711 studies identified in the search, 10 studies for the systematic review and eight for the meta-analysis met the inclusion and exclusion criteria. Overall, the use of CVs showed improvements in certain sports performance indicators, being significant (*p* < 0.05) in test time and substantial in peak power that could be influenced directly by the significant reduction (*p* < 0.05) in skin temperature and indirectly by the significant improvement (*p* < 0.05) in thermal and exertional perceptual responses, without the involvement of core temperature. In conclusion, the use of CVs is a cooling technique that influences perceptual response, thermo-physiological behavior, and sports performance. However, further studies are needed to elucidate the relevance of its application to CVs.

## 1. Introduction

In the sports activity developed by recreational and/or professional athletes, the aim is to reach the limits of their physical capacities with the optimization of the physiological functioning of the organism [1]. However, heat production is associated with the basal metabolic rate, the generation and dissipation of muscular heat (which increases dramatically at the beginning of a muscular contraction and doubles during the first minutes of dynamic exercise), the vigorous intensity of the exercise, and the climatic conditions of the environment [2]. All these elements have a decisive influence on the increase of the core temperature (Tc) of the organism [2]. The increase in Tc is accelerated when exercise is performed in hot conditions, compromising physiological capacities, impairing exercise intensity, and increasing athlete fatigue, a situation that limits athletic performance [3] and is also a health risk due to increased real and perceived thermal stress [4]. The decrease in performance has been estimated to be between 0.3% and 0.9% for every Celsius degree (°C) increase in ambient temperature above 10 °C [2], and these negative effects on fatigue are also associated with exercise duration (~2% for ~6.5 min; ~7% for 30 min) [5]. It has been established that the increased Tc associated with exercise and heat stress at a critical level of 40 °C increases cardiovascular strain, reduces the maximal volume of oxygen (VO_2_ max) and increases relative metabolic rate, and adversely affects the central nervous system (CNS) functioning by altering central activation through reduced force production [6]. Thus, the additional stress provided by heat directly alters physiological biomarkers, perceptual sensations, and physiological parameters [7] used for monitoring the health and performance of athletes in the field of sports medicine. 

Consequently, any attempt to delay body hyperthermia could minimize thermal stress by maintaining muscle recruitment, which is essential to delay fatigue and avoid decreased physical performance [2]. External cooling techniques, such as ice garments or cold towels, reduce skin temperature (Tsk), whereas ice packs, cold showers, cold water immersion, or combined methods reduce Tsk, muscle temperature, and Tc [8]. However, sports-specific cooling devices are scarce [9], although they are not for the use of cooling devices in extreme work environments, such as firefighting, aviation, chemical disposal, industrial plants and military applications where significant reductions in heart rate (HR), Tsk and sweat rate have been achieved through the use of an ice vest or ice collar [10,11]. In the sports environment cooling vests (CVs) are possibly the most practical cooling method and allow the implementation of various types of cooling strategies depending on the time of application, pre-exercise “*precooling*,” and during exercise “*percooling*” [12]. In this way, heat dissipation is facilitated by lowering Tsk without lowering the temperature of the working muscles, and heat storage capacity is increased, prolonging the time during which exercise intensity can be maintained before reaching a critical upper limit of Tc [6,13].

Therefore, the aim of this study is to investigate the effect of the use of CVs, at any time of application, on regular physical activity practitioners subjected to thermal stress, evaluating the effectiveness on perceptual responses, thermo-physiological behavior, and sports performance. Our research question was defined using the PICO model according to the standard methods proposed by the Preferred Reporting Items for Systematic Reviews and Meta-Analyses Guidelines (PRISMA) [14] as follows: P (population): physically active individuals (without any chronic disease); I (intervention): use of the CVs as a cooling device in hot situations; C (comparators): placebo/control group (CG) or pre/post comparison data group in the same conditions with/without the use of the CVs; O (outcomes): perceptual (thermal sensation [ThS]); thermal comfort [ThC]); rating of perceived exertion [RPE]), thermo-physiological (core temperature [Tc]); rectal temperature [Tre]); skin temperature [Tsk]; heart rate [HR]), sports performance (time-trial exercise; lactate concentration [LA]); maximum power [MxPO]. These biomarkers were included as meta-analysis results as they are routinely investigated in studies of health and performance markers in sports medicine research. 

## 2. Materials and Methods

### 2.1. Search Strategy

We established a structured search via the electronics databases Medline (PubMed), Web of Science (WOS), Excerpta Medica Data Base (EMBASE), Science Direct, Sportdiscus, y Scopus for studies published from database inception to 15 December 2022, restricted to English and Spanish. The terms used in the primary search were related to the use of CVs under thermal stress in healthy, physically active subjects. The search strategy contained a mix of Medical Subject Headings (MeSH) and free words for key concepts related that included: “cooling vest” OR “ice vest” OR “torso cooling” OR “precooling” OR “percooling” AND “physical activity” OR “exercise” OR “sports athletes” AND “physiological effects” OR “physiological strain” OR “thermo-physiology” OR “heat” OR “body temperature” OR “thermoregulation” OR “hot temperature” OR “perceptual responses” OR “perceived exertion” OR “core temperature” OR “skin temperature” OR “thermal comfort” OR “sports performance.” Titles and abstracts were independently reviewed by two reviewers, and the full texts of relevant articles were searched. Inclusion criteria were independently assessed, and disagreements were resolved by a third reviewer. Additional records were obtained through the reference lists of relevant included articles. Second, to account for the grey literature, the same terms as the main search on the Research Gate (www.researchgate.net, accessed on 7 July 2022) were used.

### 2.2. Selection Criteria 

We based the selection of records on the following criteria: (a) healthy adults without any chronic disease practicing physical activity, excluding animal and in vitro studies; (b) studies evaluating the use of CVs under heat stress conditions; (c) clinical trials, randomized and non-randomized trials, and pre-test/post-test design studies (excluding editorial records, reviews, notes and any other non-original studies); (d) studies that assessed as outcomes (primary, secondary) any of the perceptual, thermo-physiological and/or sports performance parameters; (e) studies with clear information on the model, duration, timing of CVs application and heat stress conditions; (f) studies of methodological quality ≥5 points according to the Physiotherapy Evidence Database (PEDro) scale; (g) no criteria related to the fitness level, sex or age of the subjects were included. Records that did not meet the above criteria were excluded from this systematic review.

### 2.3. Quality Assessment

The quality of the studies was evaluated using the PEDro scale. The PEDro scale was developed by the Physiotherapy Evidence Database to determine the quality of clinical trials [15]. This scale is based on 11 items, the first of which refers to external validity and the remaining 10 to internal validity and the presentation of the statistical analysis. Each study was awarded one point for each criterion met, while no point was awarded if the criterion was not met. The relationship between the score and the quality of the study was based on the following terms: excellent quality (9 or 10 points), good quality (6 to 8 points), acceptable quality (4 or 5 points) and poor quality (less than 4 points) [15]. 

The review protocol is published in the Prospective Registry of Systematic Reviews (PROSPERO); reference CRD42022362749. 

### 2.4. Data Extraction

Two reviewers reviewed and synthesized the data from all selected studies comprehensively in one table using standardized data extraction. Disagreements were resolved by a third reviewer. Information extracted from the selected studies included: the first author’s name, year of publication, the country in which the study was conducted, study design, sample size, participant characteristics, CV characteristics, environmental conditions, cooling strategy, parameters assessed, and outcomes.

### 2.5. Meta-Analysis Data Analysis

Firstly, we proceeded to identify and quantify the heterogeneity of our data via Cochran’s Q test and the I^2^ statistic. A *p*-value < 0.05 in the Q-test was considered proof of the rejection of the null hypothesis regarding the homogeneity of the experiments. Additionally, I^2^ values over 25%, 50% and 75% were selected to represent low, moderated, and high heterogeneity, respectively. Based on the results of these heterogeneity tests, we performed a fixed-effect meta-analysis when the absence of heterogeneity was proved. Otherwise, a random-effects meta-analysis model was employed. The variance among the studies in the random effects meta-analysis, also known as tau squared (τ2), was calculated using DerSimonian-Laird’s method [16]. The effect size (ES) was estimated as the logarithmic transformed Ratio of Means (ROM) of the CVs and placebo groups. A z-test was implemented to determine the significance of the ES. Finally, a publication bias analysis was performed using funnel plots; these graphics’ asymmetry was quantified employing Egger’s regression [17]. This bias analysis was carried out via the “Trim and fill” method. All the meta-analysis workflow was performed using the metaphor package (version 2.1-0) in R (The R Foundation for Statistical Computing, Vienna, Austria).

## 3. Results

### 3.1. Study Selection

**Figure 1:**
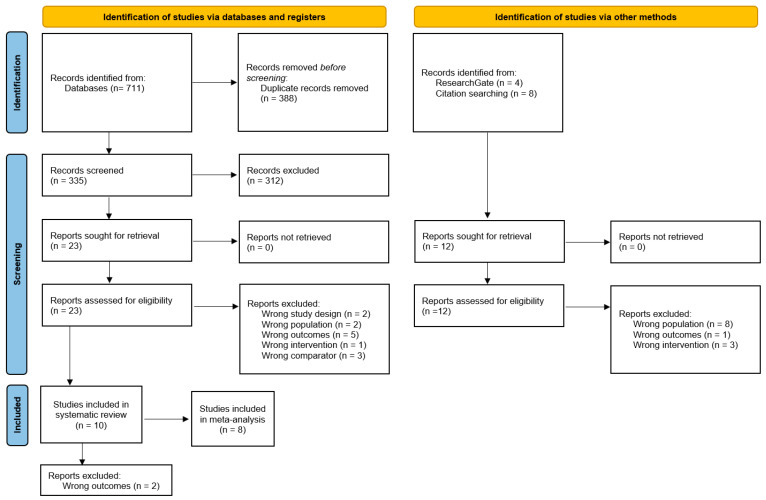
Flow diagram depicting the identification and selection processes of relevant studies according to Preferred Reporting Items for Systematic Reviews and Meta-Analyses (PRISMA) guidelines.

The literature search resulted in the finding of 723 studies. Among these records, 711 were obtained from Medline (PubMed), WOS, EMBASE, Science Direct, Sportdiscus, and Scopus and 12 from additional sources such as ResearchGate and reference lists of relevant studies. After the exclusion of 388 duplicates, a total of 335 articles were examined. Of these 335 articles, 312 were excluded for different reasons: 215 after analysis of titles and/or abstracts, 41 studies for being an inappropriate document type, and 56 articles for being unrelated to refrigeration and exercise. Twenty-three articles were considered as potential records, which underwent further full-text evaluation. Finally, a total of 10 studies were included in the qualitative synthesis (systematic review) [5,6,18,19,20,21,22,23,24,25] and eight studies for the quantitative synthesis (meta-analysis) [5,6,19,20,22,23,24,25] (Figure 1). 

### 3.2. Quality Assessment

Table 1 details the results of the criteria evaluated using the PEDro scale, where the main deficiencies found in methodological quality are associated with items 3, 5, 6 and 7 of the questionnaires. All the studies met the minimum quality score (≥5 points), reaching an average of 7 on the PEDro scale, which corresponds to good quality [5,6,18,19,20,21,22,23,24,25]. 

### 3.3. Characteristics of the Participants and Interventions 

Ten studies [5,6,18,19,20,21,22,23,24,25] included in this systematic review provide a total sample of 113 participants (81 men; 32 women), of which 77 were competitive athletes [5,18,20,21,22,25], 26 were amateur-level trained subjects [6,19,23], and 10 were recreational physical activity practitioners [24]. The application of CVs was in warm-up [5,20,22,23], warm-up and rest time [18,21], during exercise [19], recovery [6,25], or both [24] in different athletic disciplines such as athletics [20,21,22,23], triathlon [5], judo [25], and soccer [6,18,19]. The studies were performed under heat stress conditions with temperatures close to 30 °C [20,25], a range of 30–35 °C [5,6,18,19,22,23,24] and >35 °C [21] with humidity conditions < [5,6,19,21,22,23] or > [18,20,24,25] than 50% (Table 2). 

### 3.4. Evaluation of the Results of the Studies Included in the Qualitative Synthesis Systematic Review (n = 10 Included Studies)

Table 3 analyzes the information relevant to the data obtained from the study sources: first author, year and country of publication, study design, participants (baseline sample size and characteristics), cooling vest, environmental conditions, and cooling strategy.

Table 4, Table 5 and Table 6 include the main results of the perceptual responses, thermo-physiological behavior, and sports performance, respectively, of the 10 studies [5,6,18,19,20,21,22,23,24,25] included in the systematic review.

#### 3.4.1. Perceptual Response (n = 9 Included Studies)

Nine studies [5,6,19,20,21,22,23,24,25] have evaluated the effects of CVs on perceptual responses. RPE is the most analyzed parameter in the selected studies; only one study [5] showed no differences with respect to the control group (GC). The rest of the studies showed substantial [6,20,22,23,24] or significant (*p* < 0.05) differences [19,25] improvements after the use of CVs on RPE. In this sense, significant (*p* < 0.05) improvements were also observed in ThC [5,6,24], ThS [6,19,23] and humidity perception [21]. 

#### 3.4.2. Thermo-Physiological Behavior (n = 10 Included Studies)

Ten studies [5,6,18,19,20,21,22,23,24,25] included in the systematic review evaluated thermo-physiological behavior, those related to temperature changes, with different measurements being the most studied. The decreases in Tc in the experimental CVs condition were substantial [5,20,23,24,25] and/or significant (*p* < 0.05) [6,22] compared to CG. In addition, significant (*p* < 0.05) decreases in Tre [18,19], Tsk [6,18,21] and esophageal temperature (Tes) were reported [22], non-significant decreases in Tre [6,19,21,22] and Tsk [5,19,22,23] were also observed. In the evaluation of sweating, Webster et al. [21] reported significant improvements (*p* < 0.05), and four studies [5,6,19,24] showed a beneficial trend in the use of CVs versus the condition without CVs. In addition, a significant (*p* < 0.05) decrease in heat storage was observed [18].

HR showed improvement in the CVs group versus the CG in all qualitative synthesis studies [5,6,18,19,20,21,22,23,24,25], being significant (*p* < 0.05) for three studies [6,22,25]. Carballeira et al. [25] described non-significant improvements in steroid hormonal behavior analyzed by dehydroepiandrosterone (DHEA), cortisol and DHEA/cortisol ratio between judokas who apply the CVs and those who integrate the CG.

#### 3.4.3. Spots Performance (n = 9 Included Studies)

Improvements in sports performance were observed in nine studies [5,6,19,20,21,22,23,24,25] being of special consideration those that were significant (*p* < 0.05) for time-trial exercise [5,21,22], MxPO [5], VO_2_max in subjects using CVs compared to CG. However, no significant differences in LA were observed in both conditions [6,22]. 

### 3.5. Evaluation of the Results of the Studies Included in the Synthesis—Meta-Analysis—(n = 8 Included Studies)

#### 3.5.1. Perceptual Response (n = 8 Included Studies; n = 3 Outcomes)

**Figure 2:**
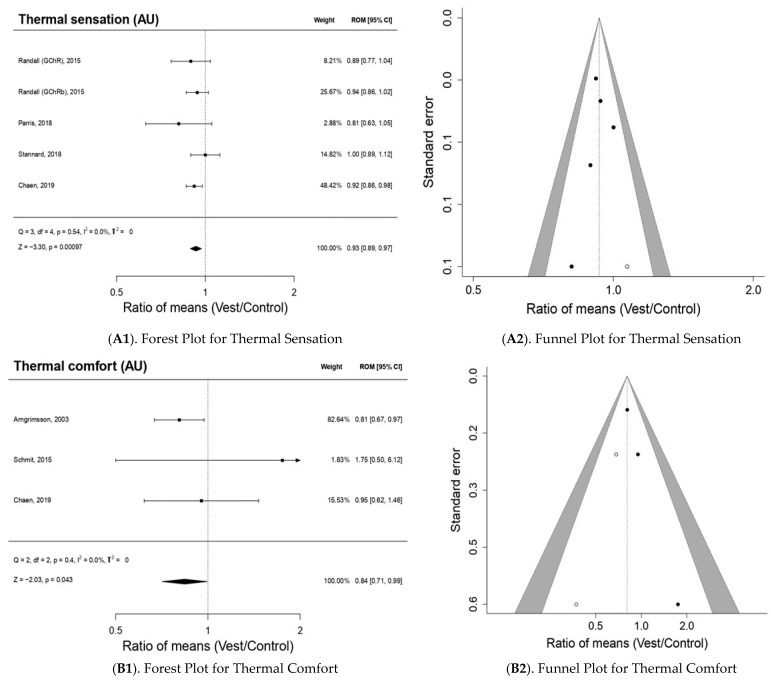

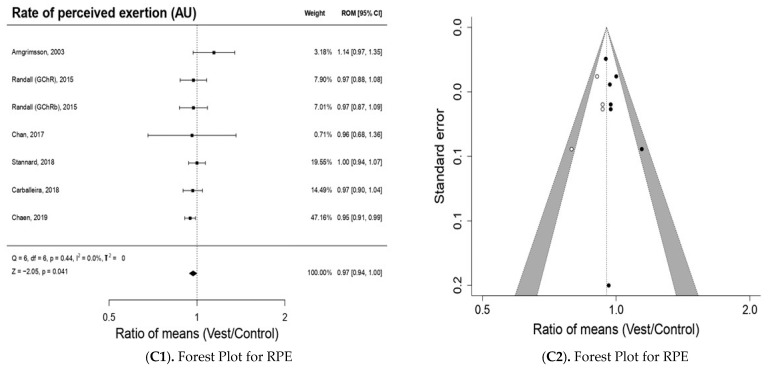
The results of the meta-analysis for perceptual outcomes.

Figure 2 shows the effect of using the CVs on the perceptual response. With respect to ThS (Figure 2(A1)), a statistically significant reduction effect is produced (*p* < 0.05): ROM 0.93; 95% CI, 0.89–0.972; Z = −3.30; *p* = 9.7×10^-04^ for the studies [6,19,20,23] analyzed in the meta-analysis. The publication bias analysis (Figure 2(A2)) for wind chill presented a relatively symmetric funnel plot, with one study being imputed that could indicate the presence of publication bias, although this asymmetry was not statistically significant (Egger *p*-value = 0.628).

The results of the meta-analysis [5,6,22] report significant improvements (*p* < 0.05) on ThC: ROM 1.01; 95% CI, 1.00–1.0; Z = −2.03; *p* = 0.043 (Figure 2(B1)). However, Schmit et al. [5] reported increases in thermal discomfort but presented great heterogeneity because of its wide 95% confidence interval (0.5–6.12). In this analysis, no significant publication bias was detected by Egger regression (*p*-value = 0.176), although the Trim and fill method did impute two studies at levels higher than ES and low standard error, which could indicate a possible lack of studies at this level (Figure 2(B2)).

In the included studies [6,20,22,23,24,25], decreases in RPE with statistically significant changes (*p* < 0.05) are observed: ROM 0.97; 95% CI, 0.94–1.0; Z = −2.05; *p* = 0.041 (Figure 2(C1)). Only one study [22] showed a slight increase in athletes with a relatively low weight (3.18%) over the meta-analysis as a whole. In this analysis, no publication bias was detected by Egger’s regression (*p*-value = 0.170); through the Trim and fill method, three studies were imputed at levels higher than ES and low standard error, which could indicate a possible lack of studies at this level (Figure 2(C2)).

#### 3.5.2. Thermo-Physiological Behavior (n = 6 Included Studies; 4 Outcomes)

**Figure 3:**
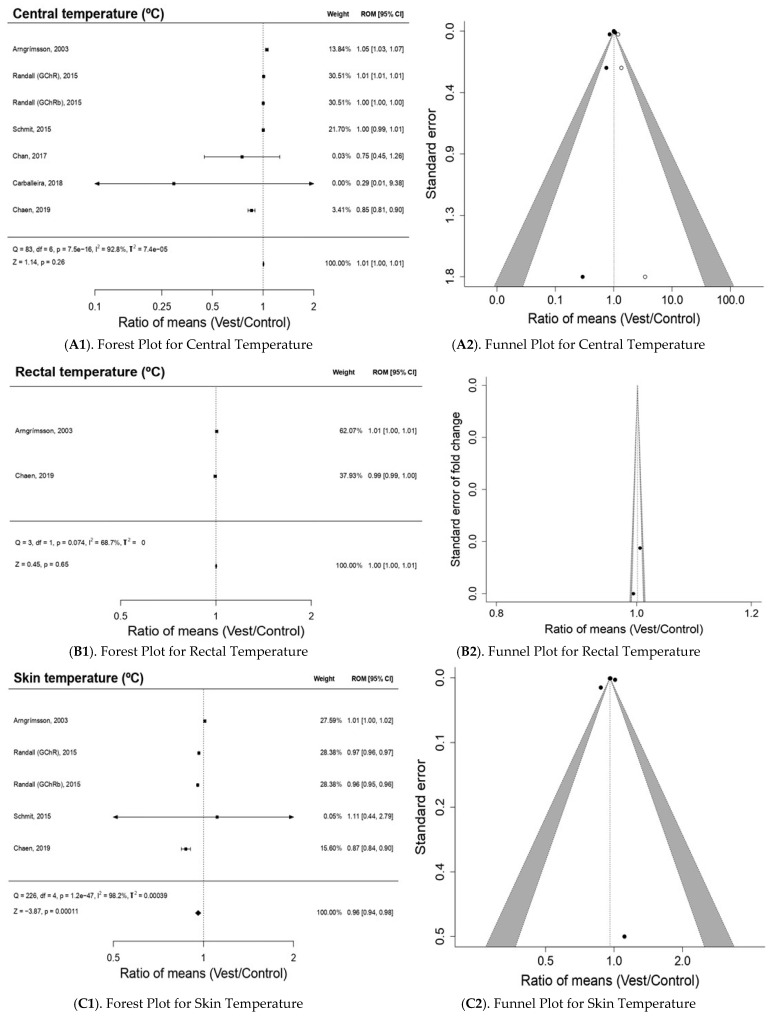

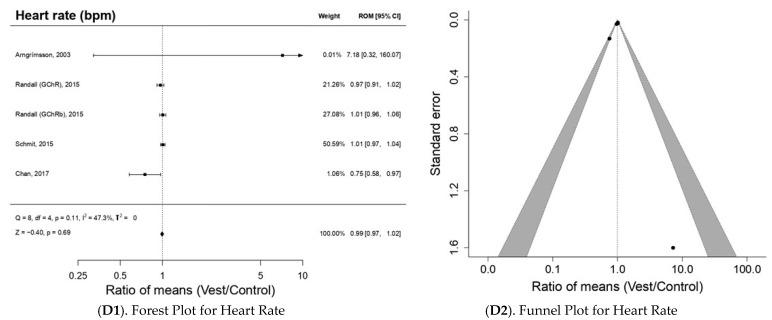
The results of the meta-analysis for thermo-physiological outcomes.

Figure 3 shows the effect of the use of CVs on thermo-physiological behavior. The use of CVs caused a significant thermal decrease (*p* < 0.05) on Tsk (*p* = 1.1 × 10^−4^), a minimal increase without statistical significance (*p* > 0.05) on Tc (*p* = 0.26) and did not change Tre (*p* = 0.65). The results of the meta-analysis of the studies that analyzed Tc [5,6,22,23,24,25] showed ROM 1.01; 95% CI 1.00–1.01; Z = 1.14; *p* = 0.26 (Figure 3(A1)); In this analysis, a publication bias was detected by Egger regression (*p*-value = 0. 0028), through the Trim and fill method there was the imputation of three studies at levels higher than ES and low standard error, which could indicate a possible lack of studies at this level (Figure 3(A2)).

Two studies evaluated Tre [6,22] with ROM 1.00; 95% CI 1.00–1.01; Z = 0.45; *p* = 0.65 (Figure 3(B1)). The publication bias analysis (Figure 3(B2)) for Tre presented a relatively symmetrical funnel plot with no presence of publication bias (Egger *p*-value = 0.0737).

Although we have reported a significant (*p* < 0.05) reduction in Tsk ROM 0.96; 95% CI 0.94–0.98; Z = −3.87; *p* = 1.1 × 10^−04^, two studies [5,22] showed results of increased Tsk; one of them [5] showed large heterogeneity with a wide 95% confidence interval (0.44–2.79) and virtually no weight (0.05%) in the meta-analysis (Figure 3(C1)), and the publication bias analysis (Figure 3(C2)) for Tsk presented a relatively symmetrical funnel plot with no presence of publication bias (Egger *p*-value = 0.1587).

Figure 3(D1) indicates that the use of CVs has a non-significant (*p* > 0.05) minimal reduction effect (ROM 0.99; 95% CI 0.97–1.02; Z = −0.40; *p* = 0.69) on HR. Arngrimsson et al. [22] showed a significant increase (*p* < 0.05) in HR but presented great heterogeneity with a wide 95% confidence interval (0.32–160.07) and practically no weight (0.01%) in the meta-analysis. In this analysis, no publication bias was detected by Egger regression (*p*-value = 0.5339); through the Trim and fill method, there was no imputation of studies at levels higher than ES and low standard error (Figure 3(D2)).

#### 3.5.3. Sports Performance (n = 5 Included Studies; n = 3 Outcomes)

**Figure 4:**
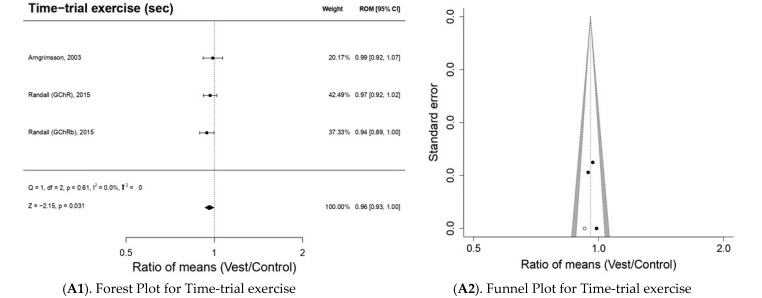

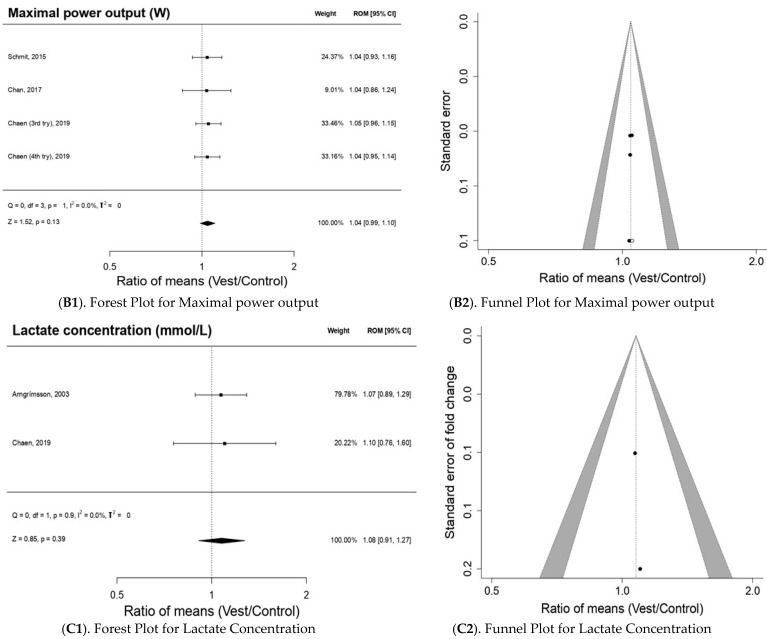
The results of the meta-analysis for sports performance outcomes.

Figure 4(A1) shows that the use of CVs produces a significant decrease (*p* < 0.05) in time-trial exercise ROM 0.96; 95% CI 0.93–1.00; Z = −2.15; *p* = 0.031. The studies analyzed [22,23] show improvements in performance with decreases in time-trial exercise. In this analysis, no publication bias was detected by Egger’s regression (*p*-value = 0.5269); however, through the Trim and fill method, there was an imputation of one study at levels higher than ES and low standard error, which could indicate a possible lack of studies at this level (Figure 4(A2)).

The use of CVs produces a minimal non-significant increase (*p* > 0.05) on MxPO: ROM 1.04; 95% CI 0.99–1.10; Z = −1.52; *p* = 0.13 (Figure 4(B1)), which would indicate a slight improvement in sports performance. All three studies [5,6,24] reported improvements in MxPO. In this analysis, no publication bias was detected by Egger regression (*p*-value = 0.9220); through the Trim and fill method, there was an imputation of one study at levels higher than ES and low standard error, which could indicate a possible lack of studies at this level (Figure 4(B2)).

Substantial, although not significant (*p* > 0.05), increases in LA were observed after the use of CVs: ROM 1.08; 95% CI 0.91 to 1.27; Z = 0.85; *p* = 0.39 (Figure 4 (C1)). In this analysis, neither publication bias analysis by Egger regression was possible due to the low number of studies nor the imputation of studies by Trim and fill (Figure 4(C2)).

## 4. Discussion

A total of 10 studies were identified in the literature for the systematic review and eight studies for the meta-analysis that met the inclusion/exclusion criteria. In general, significant improvements were observed in certain biomarkers of perceptual thermal and exertion sensations, thermo-physiological body assessment and sports performance indicators. However, the results could be influenced by the type of exercise, duration, and timing of the CV intervention. In addition, participant characteristics such as age, gender, ethnicity, body composition, training level, differences in training, nutrition, health status, and individual physiological responsiveness to cooling may also have influenced the results.

Since the 1980s, research has been conducted to reduce the temperature of the central or core zone (produces heat) and the superficial or peripheral zone (regulates heat loss) before and/or during physical exertion [26]. Recently, this research has become relevant because the most important sporting competitions on the planet have been held (World Athletics Championships 2019 Doha; Olympic Games Tokyo 2021; Tour France 2022; Football World Cup Qatar 2022) in territories with environmental conditions of high temperatures, high humidity levels and in summer, subjecting athletes to considerable thermal stress. These environmental situations pose a problem regarding thermoregulation mechanisms, which is aggravated when the individual must perform their sporting activity by inducing the formation of a very significant amount of metabolic heat, increasing the physiological stress and putting the performance and health of the athlete at risk [27]. 

Whole-body cooling techniques, using air currents or cold water baths, have been considered advantageous cooling interventions due to improvements in thermal, physiological and sports responses [28,29]. However, the logistics of moving the equipment and the need for access to water and electrical sources could pose problems [29]. In addition, the use of body zone cooling tools has also reported improved physiological and performance benefits [30,31]. It has been determined that a wide body cooling surface coverage reduces the thermo-physiological load of the organism and increases sports performance [32]. In this sense, CVs are tools that impact a relatively large body surface area and over a larger surface area than other partial/regional cooling systems [24]. The use of CVs is a strategy of an aggressive nature, which has been shown to be more effective than other local precooling and/or percooling practices [12]. The conditions of simple use of the CVs, its high level of permissiveness in the realization of training/competition practices, the avoidance of cooling of the active musculature of the exercise, the different alternatives of cooling, aesthetics, perfect coupling to the torso of the user and low weight, make it a suitable tool for subjects practicing physical activity [21]. In addition, external cooling techniques (cold towels, cold water immersion of body parts, CVs and pants) or internal cooling (ice ingestion) offer similar physiological, perceptual and sports performance responses [7]. However, combinations of cooling techniques, i.e., a mixed method of several cooling tools, had a significantly greater effect than individual cooling tools [32]. CVs provide a mixed or hybrid mechanism. The first of these is evaporative cooling which consists of reducing body temperature by evaporating sweat through the garment. This is due to the composition of the CV, which is made of a mixed fabric that includes sheep’s wool and synthetic fabric [33]. Textiles made from natural fibers, such as cotton, show a high capacity for water absorption, which can help quickly alleviate the feeling of humidity. The absorption capacity of synthetic fibers, such as polyester, is lower than natural fibers; however, they have better moisture transport than natural fibers to carry water to the textile surface for faster evaporation. This makes both fibers serve to provide a feeling of dryness to people and can potentially offer a larger surface area for evaporation [34]. The other cooling system of the vest is conduction, which is the transfer of heat by direct contact from one object to another, that is, from the body surface to the ice artifacts. Therefore, the rates of heat loss mediated by the CV will depend on the fabric and the conductivity of the material with which it is in contact [6].

The human organism has a very adjusted working temperature, and the equilibrium systems (both for heat formation and elimination) achieve their objective in a constant and continuous manner; however, situations of thermal stress, and during intense and/or prolonged exercise compromise the thermoregulation mechanisms [3]. Excessively elevated Tc has a negative impact on the ability of the CNS to generate an adequate motor impulse, reducing neuromuscular recruitment [35], force production and voluntary activation [36], and increasing alterations in metabolic processes [37] and biomarkers of muscle damage and inflammation [38]. The meager increases in Tc observed in our meta-analysis are coincident when regional hand-cooling devices and/or CVs are used [39]. 

CV mostly produces torso cooling; however, Tc reduction has been observed to be more effective when the head and/or neck are exposed to cooling systems because of their anatomical proximity to the thermoregulatory center, their better ability to perceive whole body temperature, and their superior alliesthetic thermo-sensitivity compared to other body structure during the cooling process [40,41]. Therefore humans, as a homeothermic species, have elevated Tc, which would limit exercise performance and impair health by altering thermo-physiological functions and perceptual reactions [3,27]. This implication of Tc could be even more pronounced since a possible publication bias was observed in the funnel plot through the Trim and fill method when three studies were imputed at levels higher than ES and low standard error, which could indicate a possible lack of studies at this level. However, the results of improvements in sports performance obtained in this study could reveal the non-intervention of Tc on performance, given the lack of effect of CVs on Tc. This is in line with those reported by Bongers et al. [12], who found no relationship between Tc and physical performance in heat using precooling and/or percooling techniques.

In our study, we have described the significant reduction of Tsk in the meta-analysis with a relatively symmetrical funnel plot without the presence of publication bias. Peripheral skin thermoreceptors send nerve signals to the hypothalamus (thermoregulatory center), which registers and senses elevated Tsk, modulating the intensity of physical activity so as not to exceed a critical body temperature [42]. The cooling action of CVs on Tsk could attenuate heat stress signals emitted by peripheral cutaneous thermoreceptors with hypothalamic connection [6]. This would block the signal that attenuates the intensity of physical activity, which would influence the substantial improvement in performance [5]. Achieving a cooler Tsk allows a smaller amount of cardiac output to be directed to the skin, potentially allowing greater blood perfusion to be directed to the skeletal muscle involved in physical activity [43], maintaining recruitment, muscle fiber work capacity, and maintaining desired intensities during exercise in warm conditions [32]. 

Modulation of Tsk by keeping it in lower ranges would also collaborate with thermoregulatory mechanisms, mainly by delaying the onset of sweating. Decreasing the sweating rate would ensure adequate blood volume and body water conservation protecting from the detrimental consequences of dehydration, such as increased HR, decreased blood pressure and decreased blood flow to active muscles and skin, which would lead to a significant decrease in physical performance [44]. Cooling has been reported to allow the delayed onset of sweating at higher exercise intensities, reducing HR with lower Tsk [43]. Our meta-analysis results show a reduction in HR without publication bias, which would provide optimal blood flow to meet the energy demands of exercise, thereby, at the same level of relative exercise intensity, lower heart rates. Other consequences of peripheral cooling of the CVs could have produced peripheral vasoconstriction affecting HR without any effect on Tc [25] and, in addition, visceral cooling preserving hepato-splanchnic blood flow that decreases during physical activity under heat stress conditions [7]. 

The decrease in Tsk by the use of CVs would ensure a greater temperature gradient between the core and the skin, which would drive heat dissipation from deeper regions of the body [6]. This heat transfer by conduction between the core and skin would have ergogenic potential on perceptual reactions that would increase exercise capacity and increase time to fatigue at desired intensities [45]. Visceral cooling also contributes to a lower perception of thermal stress [46]. Given that the CNS is involved in decreased athletic performance in hot conditions [7]. Perceptual enhancement adds to the sensory information from the CNS subjective elements that can qualify the sensation.

In this sense, we have reported, in the meta-analysis, significant improvements in thermal perceptual sensations (ThS and ThC) and RPE by torso cooling with CVs, which could imply its beneficial influence on the active and integrative process of heat in which the whole CNS participates, which serves to regulate effort and protect the organism from damage that could be caused by overexertion in physical activity [47]. Thus, cooling using CVs beneficially affects ThS and ThC and decreases RPE through the mediation of the motor cortex that modulates/relieves thermal stress, which is sent from the peripheral thermoreceptors to the hypothalamus [6], resulting in better performance, as we have described in the results included in our study. Improved RPE is essential in exercise tolerance and may be an indirect stimulator of sports performance indicators [48]. Thus, decreases in RPE with statistically significant changes could influence performance; even more pronounced, as a possible publication bias was observed in the meta-analysis when three studies were imputed, which would indicate a lack of studies for the index of perceived exertion. In general, the increased performance results (time-trial exercise and MxPO) are compatible with substantial increases in LA concentration, as described in the meta-analysis, which may be attributed to the higher workloads performed or a sports activity of increasing intensity [49]. 

The possible long-term physiological effects of the use of these cooling devices in athletes have not been studied. Because its use over long periods of time is not common [5,6,18,19,20,21,22,23,24,25], however, in some chronic diseases such as multiple sclerosis [50], which reduce fatigue associated with increased environmental and body temperature, they have been used. In this sense, it has been reported that the use of cooling strategies through Cvs during the 3 summer months (June, July, and August) for 40 min a day of Cvs, alleviated the severity of their fatigue without secondary effects that will alter the biological plasticity of the subjects [51]. This could position Cvs as tools to be used whenever athletes are going to train or compete for long periods of time. However, more longitudinal studies would be necessary to explain the physiological mechanisms, recommend its use, and avoid myths in the literature.

### Limitations and Strengths

Several limitations need to be acknowledged. First, a limited number of studies met the inclusion/exclusion criteria; however, our study approach followed the PRISMA guidelines [14], and the search was conducted using six relevant electronic databases in sports medicine, covered the gray literature, and the records were retrieved in English and Spanish, which makes us think that all the records in the literature were probably covered. In addition, the PEDro scale [15] was used for the evaluation of methodological quality, ensuring that all the selected studies met minimum quality criteria. Also, our systematic review was registered in the PROSPERO (CRD42022362749) public database. Secondly, there is a great heterogeneity of the studies in some of the results and the time of application, but this did not prevent us from performing a meta-analysis. The application of CVs in the included studies employed precooling and/or percooling, but no differences in ES on performance have been established between both times of use, and both cooling strategies achieve their effects through comparable underlying physiological mechanisms [12]. The great variability in the use of CVs warrants caution in interpreting the results; however, there is strong evidence for the health benefits of CVs in populations in non-exercise settings [10,11], and none of the studies reviewed reported thermoregulatory problems or heat illness. We included a considerable number of outcomes commonly used in sports medicine research to assess thermo-physiological, perceptual, or performance status. 

## 5. Conclusions

The significant improvements in time-trial exercise and important improvements in MxPO could be directly influenced by the significant reduction in Tsk, indirectly by the significant improvement in perceptual responses, essentially RPE, and without the involvement of Tc. However, following the results described in this systematic review with meta-analysis, further studies are recommended to evaluate the combination of aggressive cooling strategies, especially with CVs, on physiological, perceptual, and physical performance biomarkers. These future studies could further improve exercise performance under heat stress conditions, favoring a reduction of heat-related illnesses in athletes and other professionals who exercise under conditions of significant thermal stress. 

## Figures and Tables

**Table 1:** Results of the methodological quality assessment of included studies—Physiotherapy Evidence Database (PEDro).

Study	Item											Total Score
	1	2	3	4	5	6	7	8	9	10	11	
Arngrímsson et al. [22]	1	1	0	1	0	0	0	1	1	1	1	7
Carballeira et al. [25]	1	1	0	1	0	0	0	1	1	1	1	7
Chaen et al. [6]	1	1	0	1	0	0	0	1	1	1	1	7
Chan et al. [24]	1	1	0	1	0	0	0	1	1	1	1	7
Parris et al. [19]	1	1	0	1	0	0	0	1	1	1	1	7
Price et al. [18]	1	1	0	1	0	0	0	1	1	1	1	7
Randall et al. [23]	1	1	0	1	0	0	0	1	1	1	1	7
Schmit et al. [5]	1	1	0	1	0	0	0	1	1	1	1	7
Stannard et al. [20]	1	1	0	1	0	0	0	1	1	1	1	7
Webster et al. [21]	1	1	0	1	0	0	0	1	1	1	1	7

Item 1. Eligibility criteria; 2. Random assignment; 3. Hidden allocation; 4. Baseline comparison; 5. Blind subjects; 6. Blind therapists; 7. Blind evaluators; 8. Adequate follow-up; 9. Intention-to-treat analysis; 10. Comparisons between groups; 11. Point estimates and variability. A “1” indicates a “yes” score, and a “0” indicates a “no” score.

**Table 2:** Characteristics of the participants and intervention protocols of the selected studies.

Characteristics	Types	Studies
Age (range)	21–24 years	[6,22,25]
	24–30 years	[18,19,21,24]
	23–40 years	[5,20,23]
Sport Level	Competitions	[5,18,20,21,22,25]
	Amateurs	[6,19,23]
	Regular physical activity	[24]
Athletic discipline	Athletics	[20,21,22,23]
	Triathlon	[5]
	Judo	[25]
	Soccer	[6,18,19]
Cooling Strategy	Warm-up	[5,20,22,23]
	Warm-up & rest	[18,21]
	Exercise	[19]
	Recovery	[6,25]
	Exercise & Recovery	[24]
Temperature ° (grades)	30°≤	[20,25]
	30° & 35°	[5,6,18,19,22,23,24]
	35°>	[21]
Relative humidity % (Percentage)	50%≤	[5,6,19,21,22,23]
	50%≥	[18,20,24,25]

**Table 3:** Summary of studies included in the systematic review and meta-analysis investigating the effect of cooling vests on perceptual response, thermo-physiological behavior and sports performance in healthy adults.

First Author, Year of Publication, and Country	Study Design	Participants (Baseline Sample Size and Characteristics)	Cooling Vest	Environmental Conditions	Cooling Strategy
Arngrimsson et al. [22], 2004, USA	Random controlled counterbalanced trial	n: 17 (9 ♂; 8 ♀) Middle-long distance competition runners Age (mean ± SD) ♂: 23.4 ± 4.4 y; ♀: 22.1 ± 2.2 y Height (mean ± SD) ♂: 178.6 ± 4.4 cm; ♀: 167 ± 5.5 cm Weight (mean ± SD) ♂: 67.7 ± 4.2 kg; ♀: 55.9 ± 4.3 kg Body Fat (%) ♂: 7.3 ± 2.0; ♀: 17.8 ± 3.3	8 ice packs (450–500 mL) Neptune Wetsuits Australia, Smithfield West, Australia. Australian Institute of Sport for use by Australian Olympians.	Temperature 32 °C Relative humidity 50%	VG: For 38 min in the pre-exercise warm-up CG: standard plain t-shirt
Carballeira, et al. [25], 2019, Spain	Random controlled trial	n: 16 (8 ♂; 8 ♀) High-level judoka; ≥1st DAN, national championships medal in the last 2 y. Age (mean ± SD) ♂: 21.3 ± 2.8 y; ♀: 22.6 ± 1.7 y Height (mean ± SD) ♂: 172 ± 8 cm; ♀: 160 ± 7 cm Weight (mean ± SD) ♂: 73 ± 10 kg; ♀: 57 ± 6 kg	Arctic Heat Body Cooling Vest, Burleigh Heads, Australia. Weight ≈ 0.8–1 kg	Temperature 33 °C Relative humidity 50%	VG: rest periods 5 min (between exercises); post-exercise 10 min (recovery) CG: without a cooling vest
Chaen, et al. [6], 2019, Japan	Crossover randomized trial	n: 8 ♂ Soccer players Age (mean ± SD) 21 ± 1.6 y Height (mean ± SD) 174 ± 5 cm Weight (mean ± SD) 64 ± 4 kg	manufactured by Mizuno Co., Ltd., Japan Weight ≈ 1.9 kg	Temperature 26.9–27 °C Relative humidity 67–70%	VG: 14 min immediately post-exercise CG: cooling vest without refrigerants weight ≈ 1.9 kg
Chan, et al. [24], 2019, China	Random controlled counterbalanced trial	n: 10 ♂ practiced sports 3 times × week; physically active. Age (mean ± SD) 22 ± 5 y Height (mean ± SD) 171 ± 5 cm Weight (mean ± SD) 65 ± 6 kg	Cooling methods active (air) and passive (gel packs) Weight ≈ 1 kg	Temperature 33 °C Relative humidity 75%	VG: during exercise plus 40 min post-exercise (recovery) CG: without a cooling vest
Parris et al. [19], 2018, United Kingdom	Randomized crossover trial	n: 10 ♂ Soccer players; physically active Age (mean ± SD) 25 ± 2 y Height (mean ± SD) 177 ± 6 cm Weight (mean ± SD) 72.9 ± 7.6 kg	6626 M-PEV Kewl Fit Performance Enhancement; TechNiche International, (New Orleans, LA, USA) Weight ≈ 1.75 kg.	Temperature 33 °C Relative humidity 75%	VG: 45-min during exercise CG: 45-min during exercise without a cooling vest VG and CG: 15 min seated rest in cool conditions (23 °C, 50% humidity)
Price et al. [18], 2009, United Kingdom	Random controlled trial	n: 8 ♀ Elite Soccer players; >10 h × week^–1^, 1 match × week^–1^ Age (mean ± SD) 24.5 ± 5.1 y Height (mean ± SD) 168.1 ± 4.5 cm Body Fat (%) 16.7 ± 1.7 VO_2_max (mL × kg^−1^ × min^−1^) 50.2 ± 2.5	ArcticHeat, Burleigh Head, Queensland, Australia.	Temperature 30.6 ± 0.2 °C Relative humidity 63.5 ± 2.1%	VG: 20 min pre-exercise or both pre-exercise and during the 15 min rest period CG: no-cooling without cooling vest
Randall et al. [23], 2015, United Kingdom	Random controlled counterbalanced trial	n: 8 ♂ Highly trained athletes Age (mean ± SD) 34.8 ± 4.4 y Height (mean ± SD) 179.4 ± 4.6 cm Weight (mean ± SD) 72.0 ± 8.8 kg VO_2_max (mL × kg^−1^ × min^−1^) 65.5 ± 3.9	Arctic Heat Products, (Westwood, NJ, USA) Weight ≈ 2.4 kg.	Temperature 32.2 ± 0.8 °C Relative humidity 48.6 ± 6.7%	VG: 30 min pre-exercise, during warm-up CG: T-shirt with neutral temperature packs
Schmit et al. [5], 2015, France	Random controlled counterbalanced trial	n = 13 ♂ Well-trained national-level triathletes Age (mean ± SD) 31 ± 4 y Height (mean ± SD) 179.5 ± 4 cm Weight (mean ± SD) 71.7 ± 5.6 kg	CryoVest^®^, CryoInnov, Saint Grégoire, France. Weight ≈ 2.4 kg.	Temperature 35 °C Relative humidity 50%	VG: 25 min: passive phase (10 min) + pre-exercise warm-up (15 min). CG: without a cooling vest
Stanndard et al. [20], 2011, USA	Random controlled trial	n:7♂ Endurance runners with competitive experience (from 5 km to marathon) 12.1 ± 9.8 y Age (mean ± SD) 33.7 ± 7.4 y Height (mean ± SD) 179.6 ± 9.6 cm VO_2_max (mL × kg^−1^× min^−1^) 61.5 ± 5.8	StaCool™ Industries Inc., (Brooksville, FL, USA)	Temperature 24–26 °C Relative humidity 29–33%	VG: pre-exercise warm-up (30 min). CG: regular tight T-shirt
Webster et al. [21], 2014, New Zealand	Random controlled counterbalanced trial	n: 16 (8 ♂; 8 ♀) Competitive athletes in team sports Age (mean) ♂: 22.5 y; ♀: 20.6 y Weight (mean) ♂: 72.53 kg; ♀: 63.82 kg	A: waterproof fabric, short, close-fitting. Weight ≈ 2.80 kg B: waterproof fabric, longer. Weight ≈ 2.82 kg	Temperature 37 °C Relative humidity 50%	VG: pre-exercise warm-up (0–55 min) plus 20 min post-exercise (recovery) CG: without a cooling vest

Abbreviations n = sample size; ♂ = men; ♀ = women; kg = kilogram; y = years; cm = centimeter; min = minutes; mL = milliliters; ≈ = approximately; °C = degrees centigrade; VO_2_max = maximal volume of oxygen; wk = week; h = hour; SD = standard deviation; VG = vest group; CG = control group.

**Table 4:** Summary of the main perceptual results of studies included in the systematic review.

First Author, Year of Publication, and Country	Outcomes	Results			
Arngrimsson et al. [22], 2004, USA		**VG vs. CG**			
	RPE	†			
	ThC	†			
Carballeira, et al. [25], 2019, Spain		**VG vs. CG**			
	RPE	#			
	WI	†			
Chaen, et al. [6], 2019, Japan		**VG vs. CG**			
	RPE	†			
	ThC	#			
	ThS	#			
Chan, et al. [24], 2019, China		**VG vs. CG**			
	RPE	†			
	ThC	#			
Parris et al. [19], 2018, United Kingdom		**VG vs. CG**			
	RPE	#			
	ThS	#			
Randall et al. [23], 2015, United Kingdom		**Pre-Ex vs. Post-Ex**		**VG vs. CG**	
	RPE	↔ VG ↔ CG		†	
	ThS	↓* VG ↑* GC		#	
Schmit, et al. [5], 2015, France		**Pre-Ex vs. Post-Ex**		**VG vs. CG**	
	RPE	↔ VG ↔ CG		↔	
	ThC	↑* VG ↑* CGC		#	
Stanndard et al. [20], 2011, USA		**Pre-Ex vs. Post-Ex**		**VG vs. CG**	
	RPE	↑ VG ↑ CG		†	
	ThS	↑ VG ↑ CG		†	
Webster et al. [21], 2014, New Zealand		**VGa vs. GC**	**VGb vs. CG**		**VGa vs. VGb**
	Perception Heat	# (except the last 20 minute test)	# (except the last 20 min test)		†
	Perception Humidity	#	#		†
	Acceptability	# (20 min recovery) † (Rest of phases)	# (20 minute recovery) † (Rest of phases)		†

Abbreviations VG = vest Group; CG = control group; RPE = rating of perceived exertion; ThC = thermal comfort; WI = wellness indicator; ThS = thermal sensation; Ex = exercise; ↑*: statistically significant increase; ↑: statistically insignificant increase; ↓*: statistically significant decrease; ↓: statistically insignificant decrease; †: change without statistical significance; #: change with statistical significance; ↔: no change.

**Table 5:** Summary of the main thermo-physiological results of studies included in the systematic review.

First Author, Year of Publication, and Country	Outcomes				Results					
Arngrimsson et al. [22], 2004, USA					**VG vs. CG**					
		Tavg			#					
		Tsk			†					
		Tre			†					
		Tes			#					
		HR			#					
		Weight loss			#					
		Heat exchange rates W/m^2^		M	†					
				R	†					
				C	†					
				E	†					
				S	†					
Carballeira et al. [25], 2019, Spain					**Pre-Ex vs. Post-Ex**			**VG vs. CG**		
		Tc			↑GChR ↑*CG			†		
		HR			↔GChR ↓*CG			#		
		Hormones		Cortisol	↓GChR ↑CG			†		
				DHEA	↑GChR ↑CG			†		
				DHEA/Cortisol	↑* GChR ↑CG			†		
Chaen et al. [6], 2019, Japan					**VG vs. CG**					
		Tc			#					
		Tsk			#					
		Tre			†					
		Deep thigh temperature			†					
		HR			#					
		Sweat Rate			†					
Chan et al. [24], 2019, China					**VG vs. CG**					
		Tc			†					
		HR			†					
		PSI			†					
		Sweat Rate			†					
Parris et al. [19], 2018, United Kingdom					**Pre-Ex vs. Post-Ex**			**VG vs. CG**		
		Tre			↑*GChR ↑*CG			†		
		Tsk			↑*GChR ↑*CG			†		
		HR			↑*GChR ↑*CG			†		
		Sweat Loss			-			†		
		Sweat Rate			-			†		
Price et al. [18], 2009, United Kingdom					**Pre-Ex vs. Post-Ex**	**VGa vs. CG**		**VGb vs. CG**		**VGa vs. VGb**
		Tre			↓VGa ↓VGb ↑CG	#		#		†
		Tsk			↓*VGa ↓*VGb ↑CG	#		#		#
		Heat Storage			↓*VGa ↓*VGb ↑CG	#		#		#
		HR			↔VGa ↔VGb ↔ CG	†		†		†
		Weight			↔ VGa ↔ VGb ↔ CG	†		†		†
		Fluid Balance			↔ VGa ↔ VGb ↔ CG	†		†		†
Randall et al. [23], 2015, United Kingdom					**Pre-Ex vs. Post-Ex**			**VG vs. CG**		
		Tc			↑ VG ↑ CG			†		
		Tsk			↑ VG ↑*CG			†		
		HR			↑ VG ↑ CG			†		
Schmit et al. [5], 2015, France					**Pre-Ex vs. Post-Ex**			**VG vs. CG**		
		Tc			↑ VG ↑ CG			†		
		Tsk			↓ VG ↓ CG			†		
		HR			↔ VG ↓ CG			†		
		Plasma Volume			↑ VG ↑ CG			†		
		[Na+]			↓ VG ↓CG			†		
		Sweat Rate			↓ VG ↓CG			†		
Stanndard et al. [20], 2011, USA					**Pre-Ex vs. Post-Ex**			**VG vs. CG**		
		Tc	Warm-up		↑ VG ↑ CG			†		
			Test		↑ VG ↑ CG			†		
		HR	Warm-up		↑ VG ↑ CG			†		
			Test		↑ VG ↑ CG			†		
Webster et al. [21], 2014, New Zealand					**VGa vs. CG**		**VGb vs. CG**		**VGa vs. VGb**	
		Tre			# (Test y recovery)		†		†	
		Tsk			# (10 min test y recovery)		# (10 min test y recovery)		†	
		HR			†		†		†	
		Sweating Frequency			#		#		†	

Abbreviations VG = Vest Group; CG = Control Group; Tc = core temperature; HR = heart rate; DHEA = dehydroepiandrosterone; PSI = physiological stress index; Tsk = skin temperature; Tre = rectal temperature; Tes = esophageal temperature; Tb= Average temperature; M = metabolic heat production; A = Radiation heat exchange; C = Heat exchange by convection; E= Evaporative heat exchange; S = Heat storage; [Na+] = Plasma concentration Sodium; Ex = Exercise; ↑* = Statistically significant increase; ↑ = Statistically insignificant increase; ↓*= Statistically significant decrease; ↓ = Statistically insignificant decrease; † = Change without statistical significance; # = Change with statistical significance; ↔ = No change.

**Table 6:** Summary of the main sports performance results of studies included in the systematic review.

First Author, Year of Publication, and Country	Outcomes					Results			
Arngrimsson et al. [22], 2004, USA						**VG vs. CG**			
		Test Time				#			
		VO_2_ max				†			
		[LA]				†			
		RER				#			
Carballeira et al. [25], 2019, Spain		Manual Dynamometry				**Pre-Ex vs. Post-Ex**		**VG vs. CG**	
					Dominant Hand	↔ VG ↔ CG		†	
					Non-Dominant Hand	↔ VG ↔ CG		†	
Chaen et al. [6], 2019, Japan						**VG vs. CG**			
		Output Power				†			
		[LA]				†			
Chan et al. [24], 2019, China						**VG vs. CG**			
		Test Time				†			
		Running Distance				†			
		Output Power				†			
Parris et al. [19], 2018, United Kingdom						**VG vs. CG**			
		Sprint performance				†			
Randall et al. [23], 2015, United Kingdom						**Pre-Ex vs. Post-Ex**		**VG vs. GC**	
		Test Time (Total)				-		†	
		Test Time (Splits 0.5 Km)				-		†	
		Speed				-		†	
		Muscular Strength		MVC		↓ VG ↓ CG		†	
				VA		↓ VG ↑ CG		†	
				Quadriceps contraction strength		↑ VG ↑ CG		†	
				Maximum Amplitude		↑ VG ↑ CG		†	
				Maximum Area		↓ VG ↑ CG		†	
Schmit et al. [5], 2015, France						**Pre-Ex vs. Post-Ex**		**VG vs. CG**	
		Test Time				↑* VG ↑* VG		#	
		Output Power				↓* VG ↓ VG		#	
Stanndard et al. [20], 2011, USA		Test Time				**VG vs. CG**			
			Total (10 Km)			†			
			Splits (2 Km)			†			
Webster et al. [21], 2014, New Zealand						**VGa vs. CG**	**VGa vs. CG**		**VGa vs. VGb**
		Test Time				#	†		†
		VO_2_ max				#	#		†

Abbreviations VG = vest group; CG = control group; MVC: maximum voluntary contraction; VA: voluntary activation; VO_2_ max: maximal volume of oxygen; [LA]: Lactate concentration; RER: respiratory exchange ratio; Ex = exercise; ↑*= statistically significant increase; ↑ = statistically insignificant increase; ↓* = statistically significant decrease; ↓ = statistically insignificant decrease; † = change without statistical significance; # = change with statistical significance; ↔ = no change.

## Data Availability

All results are shown in the study.
